# Acute Hepatitis

**Published:** 1829-07-01

**Authors:** 


### MADRAS GENERAL HOSPITAL.

### Acute Hepatitis. "Morbid Matters."

The following case, related by Mr. Annesley, in the first volume of his great work on Indian diseases, offers a specimen of the severe hepatic attacks to which Europeans are liable on first entering on a tropical life in India.

"*Case.* ——Vaughan, ætat. 32, His Majesty's Royal Regiment; has only been in India two months; admitted into the General Hospital, Madras, this evening, 18th October, 1820. Says he was attacked three days ago with violent pain in his right side and shoulder; but he was either not in the way of obtaining assistance, or did not apply for it. He now complains of severe pain when he takes a full inspiration, or when the slightest pressure is made upon his side. Stools are scanty, griped, with some tenesmus; face flushed; pulse hard and full, 88; skin dry, but not hot; tongue white and furred, rather moist; much thirst; no appetite; cannot lay on either side without excessive pain, and is obliged to lay on his back day and night. He has a short, teasing cough.—Twenty leeches were immediately applied down the spine and along the right hypochondriac region, and thirty-six ounces of blood were taken from a vein.

"*Ten o' Clock*, p. m. The first cup of blood which flowed from the vein was exceedingly thick and black; the second was cupped, and had the inflammatory buff coat, with a great deal of serum; the last few ounces were of a firm, compact, flat surface, with very little serum and very little buff coat.—Calomel, gr. xx.; pulv. antim. gr. iv.; opii puri, gr. ij. h. s. s. Enema purgans. Mist. salin. febrif. every three hours.

"19th.—Has been copiously purged, and the stools are extremely offensive, feculent, and morbid; pain relieved, but still severe; pulse 84, soft and regular; skin more natural; tongue less excited.—Apply twenty leeches to the region of the liver. Pulv. purg. ʒj. Cont. mist.

"*Evening.*—Well purged, motions morbid and acrid; pain still distressing; pulse 80, full, and a sharp beat; tongue the same.—Repeat the leeches, xx.; repet. calom. et mist.

"20th.—Has not had any motion since last evening; pain considerably relieved by the leeches; tongue moist and clean; skin cool, and with a gentle perspiration; pulse 74, full, soft, and regular; he can take a full inspiration without pain, and he can lay on the left side.—Repet. enema purg. et haust. purg.; cont. salin. mist.; and apply a large poultice over the whole epigastric and hypochondriac region.

"*Evening.*—Stools extremely copious, feculent, and very morbid; is very much better; pulse, skin, and tongue, natural.—Pilul. aloë. cum calom. no. 1. three times a day. Mist. amar. cum sennâ, ℥iij. nocte maneque. Cont. poulticing, &c. &c.

"21st.—Stools scanty and dark coloured; slight pain in the side.—Pulv. jalap. comp. ʒj. and cont. omnia.

"These medicines were continued daily till the 25th, motions being always morbid, offensive, and crude; he complains this morning of return of pain.—Fifteen leeches were applied immediately; and the purgative medicines, poultice, &c. continued; and a large blister was afterwards applied on the right side.

"28th.—Mouth slightly affected; pain less; motions green, viscid, and tenacious, and very copious.—Cont. med.

"30th. Mouth very sore; considerable ptyalism.—Omit the pills. Cont. mist. amar. cum sennâ; milk diet. Blister discharging freely; pulse much accelerated; pain diminished. Cont. mist. salin., poultice, &c. &c.

"*Nov.* 3d.—Stools copious, feculent, and of a dark-yellow colour; more natural; considerable fulness about the liver, and the ribs are evidently raised considerably; has rigors.—Cont. poultice and nitro-muriatic lotion.

"6th.—Stools perfectly natural; enlargement of the liver subsiding. Cont.

"9th. Stools regular; pulse 62; ptyalism diminished; no rigors; bowels not sufficiently acted upon.—Pulv. jalap. comp. ʒj.

"11th.—Stools copious; green, viscid, tenacious matter; has more pain in his side, chiefly felt at the margin of the ribs and towards the posterior part of the liver, and it is increased on a full inspiration; has pain in his shoulder, which shoots up from the loins; no rigors or shivering; mouth still sore.—Repet. pulv. purg., and apply sixteen leeches to the posterior part of the liver. Cont. poultice and nitro-muriatic lotion.

"*Evening.*—Fully purged; stools offensive, and almost black.—Cont. haust. amar. cum sennâ; mist. salin. febrif.; poultice, &c. &c.

"13th.—Has a dull, heavy pain in the side, but not at all sharp; his mouth is quite well.—Pilul. aloë. cum calom. three times a day. Cont. omnia.

"16th.—The fulness and enlargement of the side very much reduced, but he has still a dull pain; stools improving very much.—Cont. ut anteà.

"23d.—Gums again affected.—Discontinue the pills. Cont. alia.

"30th.—Ptyalism continues; he has been gaining ground daily, and his bowels are becoming quite regular; but he had another attack of pain in the side last night, precisely similar to that which he had on the 11th.—Apply sixteen leeches. Pulv. purg. ʒj. stat. Repet. poultice and lotion.

"*Dec.* 1st.—Much better; the pain greatly relieved.—Cont.

"This system was continued for near three weeks longer, with the occasional application of leeches; and he returned to his regiment, perfectly recovered."

*Remarks.* Mr. Annesley observes on this case, that "the morbid condition of the alvine excretions was almost incredible till the last week of his convalescence." "He was regularly purged every day—and the mass of foul, morbid matter that came from him was astonishing." Mr. Annesley queries, "could this have arisen from accumulations or morbid secretions?" We think that few physicians or physiologists will hesitate to say that these masses of morbid matters were generated from day to day—not merely removed piece-meal from an Augean stable. Mr. A. adds that, as purgation cured the disease, the foregoing state of the bowels "was not caused by purging." We are not quite sure of this. We strongly suspect that a much less quantum of purgative materials might have sufficed, had the patient been kept quiet in bed, with nothing but rice-water to live upon, and leeches every two or three days applied to the hepatic and epigastric regions.

We have, at this moment, under our care, a gentleman from whose bowels we can evacuate bucketfuls of "morbid matters," at any time, by active purgation. Yet if he be confined to bed—kept on chicken or veal broth—and takes small doses of the pulvis ipecacuanhæ compos. with the hydrargyrus cum cretâ, the motions soon become consistent, and as healthy as those of an infant. These are the people who become the prey of Charlatans, when swallowing daily doses of drastic purgatives that irritate every secreting gland and surface from the mouth to the anus, and thus generate the masses of "MORBID MATTERS" which they pretend to merely eject, by means of their wonder-working cholagogues.

